# Adjustment of Root System Architecture and Root Function in Response to Multifactorial Abiotic Stress

**DOI:** 10.1111/ppl.71170

**Published:** 2026-10-29

**Authors:** Karl‐Josef Dietz

**Affiliations:** ^1^ Biochemistry and Physiology of Plants, Faculty of Biology Bielefeld University Bielefeld Germany; ^2^ CeBiTec Bielefeld University Bielefeld Germany

**Keywords:** abiotic stress, acclimation, cell signaling, multifactorial stress, root architecture, root growth

## Abstract

The root of land plants is a remarkably adaptive organ. Unlike the shoot with its main functions in light harvesting and photosynthesis, sexual reproduction and diaspore distribution, the root explores the soil for nutrient resources and water, while preferably avoiding zones of unfavorable conditions. Roots realize an extraordinarily flexible architecture and perceive and integrate environmental input parameters to optimize growth, architecture, and physiology. In nature, roots encounter a heterogeneous rooting environment characterized by combinations of negative and positive growth parameters, for example, nutrient availability, temperature, hypoxia, toxic metal ions, or compacted soil, necessitating research on multifactorial stress effects on roots. While many studies have addressed the combination of altered nutrient status, in particular nitrogen and phosphorus, and other environmental stress factors such as drought, temperature, and soil compaction, fewer studies addressed climate change‐related combinations of stressors such as heat, drought, and hypoxia. The decisive mechanism of environmental input integration targets root growth and architecture, but also exudation. The adjustment is realized by altered activity of apical meristems for root elongation, the pericycle for lateral root initiation and formation, the development of adventitious roots, and root thickening by cambial activity and senescence. This review presents selective case studies to exemplarily summarize current experimental approaches and obtained knowledge on root responses to multifactorial environmental constraints with focus on assimilate‐, redox‐, and auxin‐dependent regulation. The review pinpoints the need to advance and harmonize our experimental approaches by investigating the sensory role and growth response of roots at the local and systemic level.

## 1 Introduction

Roots of cormophytes realize nutrient and water uptake and anchor the plant. The simplicity of this statement contrasts the requirement for developing the root system in close accordance with the prevailing environmental conditions in the soil, in particular aligning growth with the resource availability, but also avoiding local areas and hotspots of chemically and physically unfavorable soil volumes (Zhang, Mu, et al. 2023; Zhang, Fang, et al. 2023; Wang et al. 2023) (Figure 1). Usually neither water and nutrients nor other abiotic parameters such as bioavailable heavy metals, oxygen and temperatures, and adverse or beneficial biotic interactors distribute evenly in the soil, for example, in upper and lower soil layers, and this heterogeneity persists in the agricultural context. Importantly, phenotyping of root architecture currently advances rapidly by using imaging technologies including nuclear magnetic and X‐ray tomography, and is assisted by machine learning approaches. This advancement allows researchers to monitor the conditional root development in unprecedented detail and link it to molecular events (Morris et al. 2017; Pasternak and Perez‐Perez 2021; Weihs et al. 2024).

**FIGURE 1:**
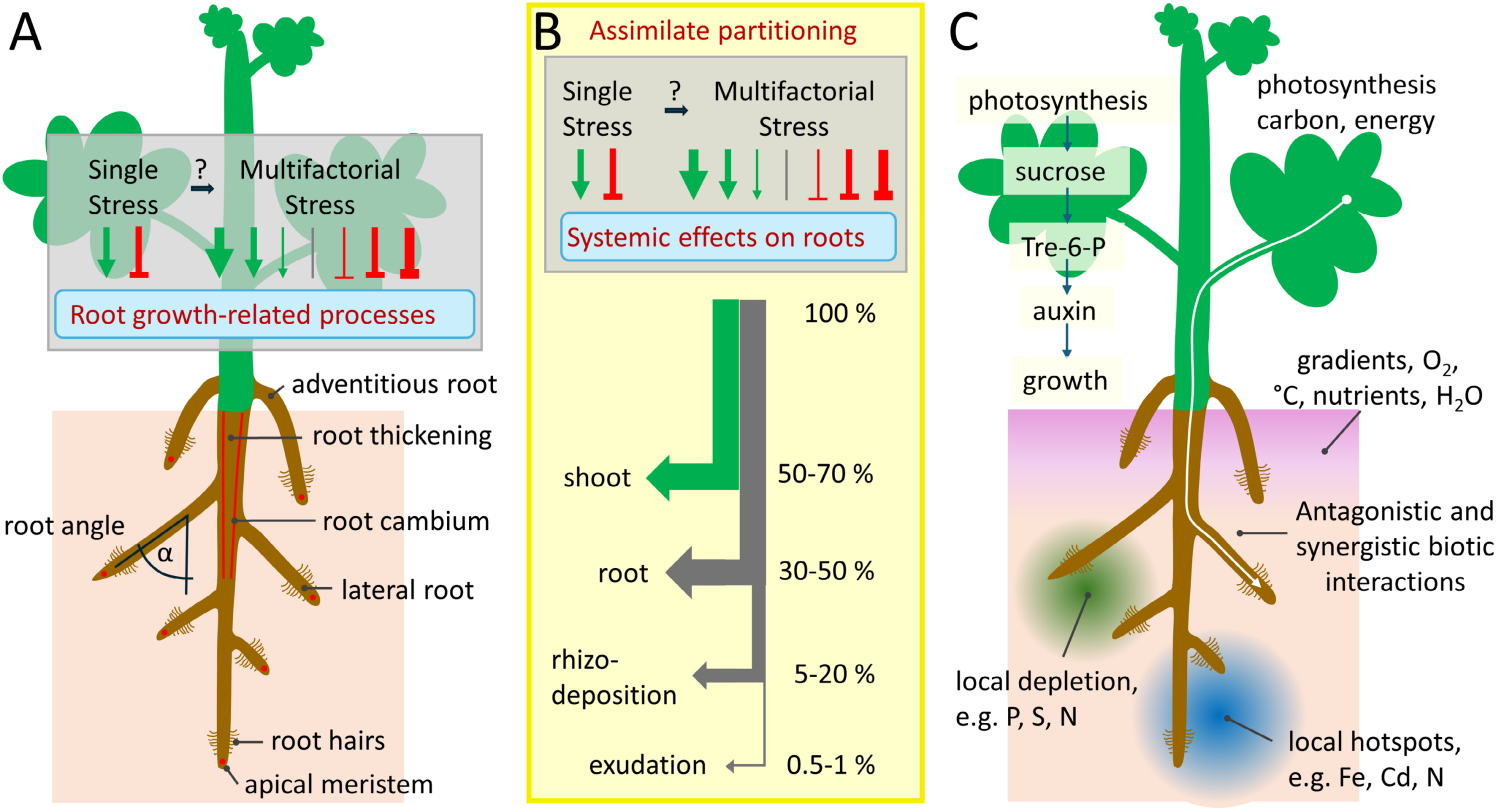
Schematics of plant and root system architecture (RSA), assimilate partitioning during plant growth and parameters of the soil environment that affect RSA formation. (A) The conditional activities of meristems and cambia determine the growth of apical, lateral, and adventitious roots, their initiation and thickening. These processes are often differently changed in response to single and combined stress, as indicated by the various forms of activating and inhibiting interactions (lines with error heads or T‐bar). (B) Long distance interactions and C‐assimilate partitioning to the root during vegetative growth constitute systemic parameters and participate in synergistic and antagonistic responses to multifactorial stress combinations. (C) Determinants of RSA formation. Systemic signals from the shoot and concentrations and gradients of chemical and physical parameters in the rooting substrate interfere with root development. Heterogeneities often exist between the top soil and deep soil layers, or in the form of local hotspots of toxins or depletion of nutrients or oxygen, but also antagonistic and synergistic interactions with the microbiome including symbiotic interactions.

This section introduces the basics of root growth and architecture, mineral nutrition and stress research. Morphologically, root architecture is shaped by the arrangement of primary, lateral, and adventitious roots (Figure 1). At the cellular and tissue level, roots of angiosperms consist of the cap, the stele, the cortex, and the epidermis (Figure 2). The root epidermis (rhizodermis) of dicots originates from stem cells together with the cap, whereas the root epidermis of monocots and some families of the Nymphaeales develops as boundary tissue of the cortex from common stem cells (Clowes 2000). The different origin coincides with differences in the patterning of root hair‐forming trichoblasts and atrichoblasts in the epidermis (Clowes 2000). This work also describes the variation in root tissue patterning in different species (Clowes 2000). Such findings have to be kept in mind when we often focus on the model plant *Arabidopsis thaliana*, which presumably has the deepest molecular dissection of the involved processes but has little relevance to agriculture and complex ecosystems.

**FIGURE 2:**
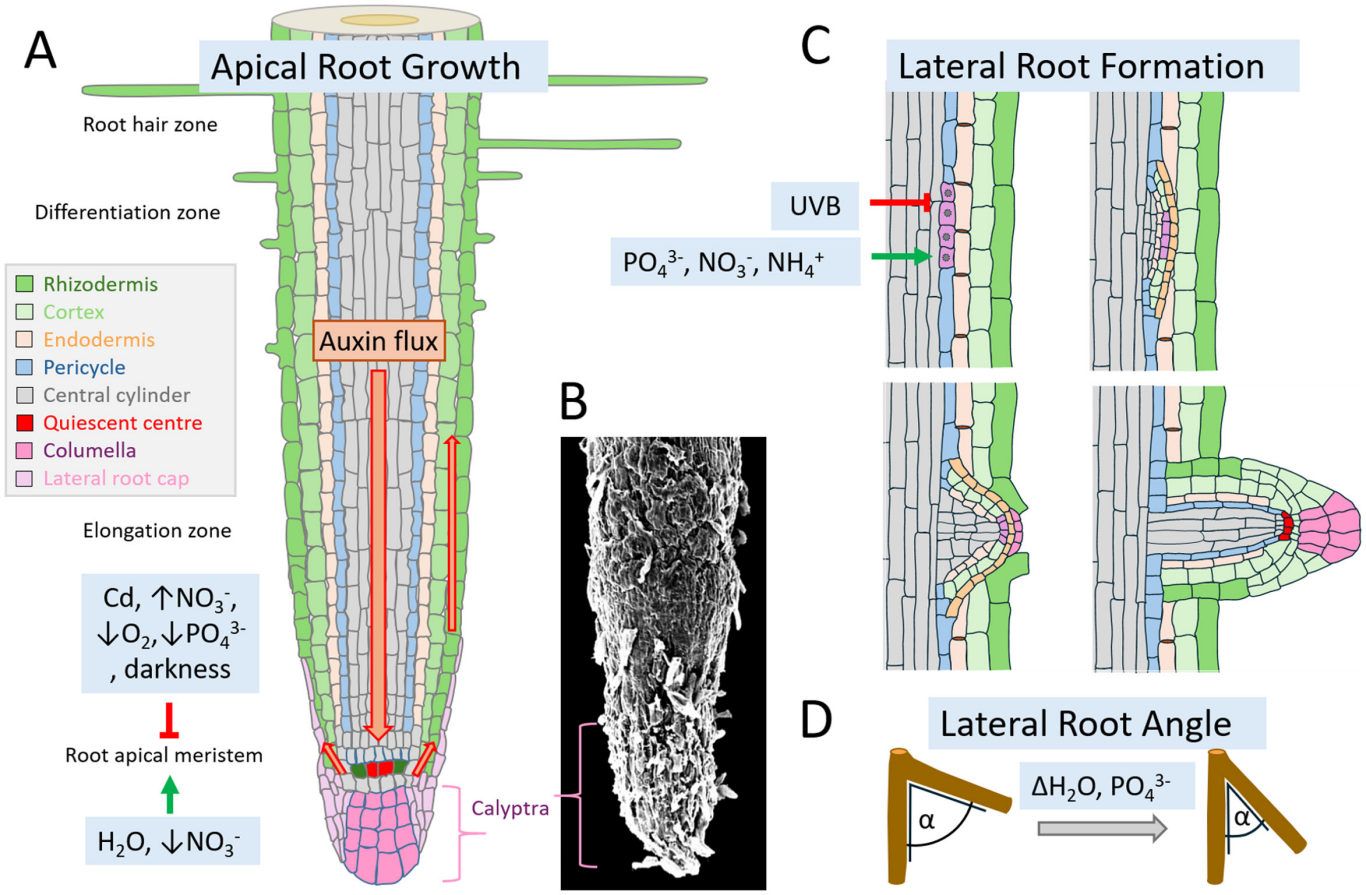
Schematics of root tip anatomy and exemplary parameters interfering with root developmental processes. (A) Schematics of the apical root with color coding of different cell types and functions. Also indicated are selected chemical parameters that inhibit or stimulate apical root growth. The arrows indicate high or low intensity or concentration. (B) Electron micrograph of the tip of a barley primary root showing cells and tissue fragments released from the roots to the rhizosphere as part of the rhizodeposition. (C) Schematics of lateral root initiation and early development and selected parameters that stimulate the process, while UVB inhibits lateral root formation. (D) Simplified depiction of lateral root bending and exemplary parameters that affect the process.

Six developmental processes determine the root architecture: (1) the cell division activity of the root apical meristems (RAM); (2) root cell elongation and differentiation, including formation of root hairs; (3) the initiation and growth of lateral roots; (4) root thickening; (5) the formation of adventitious roots; and (6) senescence. Each of these processes is under control of endogenous factors and exogenous stimuli that act locally or over long distances (De Nittis et al. 2025). Adventitious roots can emerge from different tissues. Following the development of the embryonic primary and seminal roots in *Poaceae* like *Zea mays*, first crown roots emerge as postembryonic adventitious roots from below‐ground nodes of the shoot and subsequently brace roots from above‐ground nodes (Hochholdinger et al. 2004). Adventitious root formation also contributes to the flexible root architecture of dicots and is important for crops such as potato and tomato (Joshi and Ginzberg 2021).

Plant growth depends on the six macronutrients calcium, magnesium, nitrogen, phosphorus, potassium and sulfur (de Bang et al. 2021) and at least eight micronutrients such as iron, zinc, copper and boron (Lilay et al. 2024). The nutrients become available from inorganic sources, dissolved ions or weathering of minerals, and organic sources, or are delivered by rhizosphere‐associated microorganisms, including mycorrhiza. Competition between roots of neighboring plants and other biotic and abiotic interactions strongly affect root development. As a consequence of low availability and high competition, plants often encounter nutrient deficiency in nature (de Bang et al. 2021; Lilay et al. 2024).

Likewise, heterogeneous drying and water potential gradients in soils are a common phenomenon in nature and first affect water availability in the topsoil. Despite dry topsoil, deep rooting of the plants may still allow for water uptake from deep soil layers in desertified areas and during drought, also in agriculture (Tariq et al. 2026). Therefore, water potentials in natural soils are usually heterogeneous, and this is intentionally aggravated in agriculture by water‐saving irrigation practices like drip irrigation (Li et al. 2025).

Profound knowledge of spatial and temporal responses to the environment has been developed based on global and cell type‐specific transcriptome profiling, and by combining these findings with the physiological acclimation processes of plants to nutrient deficiency or nutrient excess, soil acidity or salinity (Dinneny et al. 2008; Gifford et al. 2008; Iyer‐Pascuzzi et al. 2011). Apparently, we understand the principal mechanisms that enable the dynamic optimization of root architecture and function for acclimation or adaptation to a heterogeneous environment (Morris et al. 2017; Ramachandran et al. 2025).

To better understand plant responses to combinatorial constraints, several terms need to be introduced. Stress describes a negative environmental factor that strongly impairs plant performance. Presently, the term stress is used in a very broad sense, comprising both the environmental stress factor and the strain on the plant. If several stress factors impact a plant, the perception and signaling of each factor are integrated, often eliciting a response that differs from simple additivity, and this kind of interaction can be synergistic, antagonistic, or neutral (Dietz 2021). This review is a follow up of a special issue on root responses to multifactorial environmental constraints and relates to the Editorial (Dietz et al. 2026) and, for example, the review on Sorghum root responses in a combinatorial stress environment (Sargar et al. 2025). In the next section, this review starts out with the interaction between nitrogen availability and different abiotic stresses as important and relevant examples of well‐studied multifactorial stress.

## 2 Multifactorial Stress Scenarios Involving Nitrogen Nutrition

In many recent papers, it is argued that our knowledge on combinatorial stress impact on roots is scarce. However, there are exceptions to this statement, in particular with respect to nutrient availability combined with abiotic or biotic stresses. Many studies have explored environmental conditions where nitrogen deficiency or nitrogen excess, either provided as nitrate (NO_3_^−^), ammonium (NH_4_^+^) or as a combination of both, have been combined with other stressors such as drought (Drobnitch et al. 2026), cadmium toxicity (Wang et al. 2026), soil compaction (Mondal et al. 2022) or unfavorable temperature (Garmash 2005; Ru et al. 2024). Similar studies are available for other nutrient deficiencies, in particular with respect to phosphate deficiency in combination with drought, for example, in peanut and the mitigating role of rhizobacteria (Lopez et al. 2025), salinity in alfalfa (Su et al. 2022) or combined with arsenic toxicity in wheat (He et al. 2025). Here, we use the example of N deficiency to present selected studies on the impact of multifactorial stress.

N‐foraging, N‐uptake and N‐incorporation strongly control root growth and architecture. NO_3_^−^‐deficiency promotes apical root growth in order to extend the foraging area and inhibits lateral root initiation and expansion. Reaching a NO_3_^−^ rich soil volume stimulates lateral root formation to exploit the local N‐resource (Figure 2). The NO_3_^−^ sensors NRT1.1/NPF6.3 in the absence of NO_3_^−^ suppress auxin transport to the pericycle and inhibit lateral root formation in *Arabidopsis thaliana* (Zhang et al. 2026). If NO_3_^−^ becomes available, a protein phosphorylation cascade involving the mitogen‐activated protein kinase (MAPK) cascade, and specifically MEKK13/14, and downstream protein kinases activates auxin‐dependent lateral root formation (Zhang et al. 2026). The following sections describe case studies in which N deficiency was combined with other stressors and examined for its effects on root architecture and function.

### 2.1 Drought

The interaction between drought and nitrogen affects root system architecture and function, and it differs among functional and phylogenetic plant groups and genotypes. Moderate N supplementation supports plant growth and root development of maize, rice, and wheat under drought, while high N levels are detrimental if combined with water deficit, as derived from a meta‐analysis after standardization of the literature data (Drobnitch et al. 2025). In clay‐grown *Sorghum bicolor*, the response of roots to high versus low N in water‐limited conditions varies between genotypes. The above‐ground and total biomass of the genotypes and species are not significantly changed at low and high N under water limitation. However, they differ in their root response, expressed as accumulation of structurally important coarse roots and resource‐acquiring lateral fine roots (Drobnitch et al. 2026). Water deficit dominates the root response of the tree *Pinus koraiensis*, with a shift toward a thick, dense, and short root system. Increased N has little effect on the drought‐induced root morphology of this coniferous tree in a field study at the Research Station of Changbai in China (Tian et al. 2026). It should be noted that RSA responses to *N* × drought interactions can differ substantially between annual crops and woody/perennial species due to differences in root system development, root longevity, secondary growth, and resource‐acquisition strategies. These important additional boundary conditions cannot be covered in detail here. Nevertheless, the few examples show that the N × drought interaction strongly affects RSA and depends on genotypes and species. This interaction requires two‐sided consideration, as low‐N environments can mitigate or enhance the plant response to drought stress, and vice versa, drought stress may interfere with the plant response to N deprivation.

### 2.2 Compact Soil

Compacted soil is a common phenomenon and relates to the soil type and mechanical load. High soil density restricts root growth mechanically and impedes water and nutrient acquisition by lowered water flow. Nutrient uptake by barley decreases with increasing soil compaction in controlled laboratory experiments (Arvidsson 1999). The ability of roots to penetrate compacted soil depends on nutrient availability, species, and genotypes. The wheat genotype *Choti Lerma* grows poorly in dense soil in the presence of high nitrogen. Growth improves at low N‐supply. In a converse manner, *HD‐2967* wheat displays the same root length density in dense soil at low or high N‐availability during a 7‐week growth period in pots (Mondal et al. 2022). The nitrogen deficiency response at the gene regulatory level is enhanced in compacted soil; however, with genotypic variation.

### 2.3 Cadmium

High concentrations of heavy metals inhibit apical and lateral root growth. Interestingly, at subtoxic concentrations, effects of different heavy metals on roots vary widely (Sofo et al. 2022). *Arabidopsis thaliana* rapidly activates mechanisms to overcome cadmium‐induced root growth inhibition under high‐N conditions of 25 mM NO_3_^−^ when grown on plates (Wang et al. 2026). Asymmetric incorporation of the nitrate transporter NRT1.8 in the adaxial plasma membrane of the lateral root cap promotes nitrate transport into the epidermal cells. Subsequently, localized synthesis and translocation of nitric oxide are suggested to activate RAM activity and root growth even in the presence of toxic Cd^2+^ concentrations of 50 μM. This study provides experimental evidence that root growth inhibition by Cd^2+^ is due to a regulatory inhibition that can be overcome by antagonistic signaling and regulation involving N resource availability (Wang et al. 2026).

Toxic cadmium levels added to hydroponics inhibit apical and lateral root growth, for example, of *A. thaliana* seedlings (Li et al. 2010) and, likewise, high nitrate supply decreases root growth relative to optimal concentrations (Li et al. 2010). The extent of Cd^2+^ inhibition remains unchanged between wild‐type and the mutant *ntr1.8*, which is devoid of the NO_3_^−^‐ and Cd^2+^‐induced nitrate transporter NRT1.8 that is expressed in the xylem parenchyma cells of the root vasculature. The combined treatment with Cd^2+^ and high NO_3_^−^, however, almost completely suppresses root elongation of *ntr1.8* mutants on agar‐solidified medium (Li et al. 2010). The presence of NRT1.8 induces a response that lowers the Cd^2+^ concentration in the xylem (Li et al. 2010; Wang et al. 2026). Recent work showed that NRT1.8 mediates NO_3_^−^ uptake into the root tip and induces local synthesis of nitric oxide (NO) in the epidermal cells. Accumulating NO may stimulate meristem activity and root growth (Wang et al. 2026). The role of NO enrichment in Cd^2+^‐exposed roots is debated, since in experiments with other mutants, NO correlates with root inhibition due to altered PIN and auxin levels (Fernandez‐Marcos et al. 2011; Yuan and Huang 2016). Detailed analysis of spatial distribution and local concentrations of NO_3_^−^, NO, and NTR1.8 reveals a major role of the cells of the lateral root cap (Wang et al. 2026). Irrespective of the precise mechanisms, the studies demonstrate the role of nitrogen and NO signaling in acclimating to combinatorial NO_3_^−^‐ and Cd^2+^‐stress treatments.

### 2.4 Temperature

Temperature plays a critical role in regulating nutrient use and plant growth. Heat waves are particularly detrimental if they coincide with the developmental stages of flowering, pollination, and fruit setting. As described for the drought *×* nitrogen interaction, lowered N supply to wheat is also advantageous for mitigating the negative effects of combined heat and drought impact (Ru et al. 2024). The lowered N availability is beneficial because it triggers the development of a larger root system in this outdoor pot experiment.

Barley root growth is less affected by low temperature and low nutrient supply than shoot biomass. This experiment was conducted in a phytotron chamber in hydroponics. The root‐to‐shoot ratio at high nutrient supply rate is 0.27 at regular temperature (21/17°C day/night) and 0.31 at low temperature (13/8°C). It increases to 0.78 in plants at high temperature and low nutrient conditions, but only to 0.53 in low nutrient supply at 13/8°C (Garmash 2005). These changes inversely reflect the N/C‐ratio in the plant. At high nutrient supply, the N/C‐ratio is 0.141 and 0.139 at high and low temperature, respectively, and it drops to 0.057 in low nutrient condition at high temperature, and only to 0.088 at low temperature. These and additional growth parameters demonstrate that low temperature mitigates the growth limitation by low nutrient supply, particularly of roots (Garmash 2005). At low nutrient availability and high temperature, the roots are programmed to forage for nutrients by strong stimulation of the apical meristem(s) for longitudinal root growth at the expense of shoot development.

These selected examples demonstrate the elaborated morphological and, in part, physiological knowledge on combinatorial stress effects involving nitrogen availability in conjunction with other abiotic stresses. Similar studies on the interaction of nutrient availability and abiotic stress such as drought exist for phosphorus, potassium, and other nutrients (Bauer et al. 2025; Xu et al. 2021; De Barros et al. 2026). Many of these studies describe the phenotypic changes but scarcely address the underlying molecular mechanisms, despite the fact that the involved regulators and processes are reasonably well understood as outlined in the next section.

## 3 The Interaction of Assimilate Supply and Auxin Patterning Determines Root Growth

Prominent signals and regulators that determine root growth are assimilate availability, phytohormones, and redox‐related processes, also involving peptides and non‐coding RNAs (De Nittis et al. 2025; Gomes and Scortecci 2021). Cell cycle dynamics are closely related to root growth and differ between different cell types and cell positions (Pasternak et al. 2022). Importantly, chromatin organization and cell volume distribution can be used to study the root zonation and growth performance in the RAM zone, overcoming drawbacks of marker‐based RAM assessments (Pasternak and Perez‐Perez 2021). These authors considered the cell cycle as the ‘sum of chromatin phase transitions’ and ‘changes in nucleus structure’. Importantly, this kind of methodological approach is applicable to species that are less amenable to molecular dissection compared to *A. thaliana* and offers a manageable approach to multifactorial stress scenarios.

Environmental factors affect source tissue activity and assimilate provision from the shoot or storage organs to the roots and nutrient acquisition in the soil. Single and combinatorial stress treatments that affect photosynthesis and long‐distance transport alter RSA. Both processes interfere with hormone synthesis, transport and action and, thus, downstream signaling. Hormones, most prominently auxins, act as integrators of environmental stress input including combinatorial stress impact. Hormonal signaling bidirectionally interferes with redox and reactive oxygen species (ROS) signaling (Singh et al. 2024; Shen et al. 2025). Most environmental stress factors affect the redox/ROS state and hormonal state of tissues, and the amount of ROS indicates stress intensity (Dietz and Vogelsang 2024).

Long‐distance transport from source tissue provides assimilates through the phloem to the roots and drives root growth (Figure 1). Sugar availability and auxin are intricately connected (Mishra et al. 2022). Decisive regulators that convert sugar and energy availability into root growth are apoplastic invertases to release glucose from sucrose, trehalose‐6‐phosphate as a sugar‐related messenger (T6P), and the energy and sugar sensory system of Sucrose‐Non‐Fermenting 1 Related Protein Kinase 1 (SnRK1) and Target of Rapamycin (TOR) (Morales‐Herrera et al. 2023, 2024). SnRK1 inhibits carbon‐ and energy‐consuming pathways, whereas TOR stimulates anabolic pathways. SnRK1 and TOR antagonistically control root growth and lateral root formation and, in turn, are controlled by T6P, since local elevation of T6P immediately promotes lateral root formation. T6P inhibits SnRK1 and activates TOR, which stimulates lateral root growth (Morales‐Herrera et al. 2023). The TOR pathway determines the use of energy and carbon resources for growth, interferes with the redox state of the cell, controls protein synthesis, and affects photosynthesis (Xiong and Sheen 2014; Kumar et al. 2025).

Through this mechanism, the TOR pathway integrates energy, C‐, N‐, and S‐metabolism and hormone signaling with root growth and must be considered in the context of plant responses to multifactorial environmental stress conditions as reviewed here (Saksena et al. 2026). Under cold stress, inhibition of TOR down‐regulates translation facilitating acclimation, and this process depends on Thyroid Adenoma Associated (THADA). Accordingly, the *A. thaliana* mutant *Atthada* displays cold hypersensitivity (Dong et al. 2019). Abscisic acid accumulates under drought and inhibits root apical meristem activity. Export of SnRK1 from the nucleus to the cytosol inhibits TOR, and this mechanism is suggested as a general strategy to acclimatize to environmental shifts (Belda‐Palazón et al. 2022).

The antagonism between SnRK1 and TOR and the upstream and downstream signaling pathways that coordinate root growth also occurs in response to multifactorial stressors, as reviewed by Retzer and Weckwerth (2023). Environmental parameters that impair the plant energization and assimilate status can affect root growth through this regulatory system. Thereby, environmental stresses that exclusively or mostly affect shoot activities act as co‐stressors to the roots.

Sugar availability correlates with polar auxin transport (Poelmans et al. 2025). Establishing local auxin amounts and concentration gradients determines where roots initiate, grow and elongate (Brumos et al. 2018; Morffy and Strader 2018) (Figure 3). The polar auxin transport from the shoot and root base to the root apices occurs through the vascular cylinder. In the root tip, auxin is distributed to the cortex parenchyma and epidermis layers, from where auxin is transported basipetally to the elongation zone (Roychoudhry and Kepinski 2022). The local and asymmetric arrangements of the auxin exporting PIN FORMED (PIN) transporters and the auxin influx carriers AUX establish the auxin gradients and local auxin maxima. Eight PIN proteins (PIN1‐8), four AUX proteins (AUX1 and three LIKE AUX1 [LAX1‐3]) and other transporters determine the auxin gradients and local accumulation in *A. thaliana*. Their cell‐specific expression in the vascular tissue, pericycle, endodermis, quiescent center, cortex, epidermis, root cap and lateral root cap controls the centrally acropetal auxin flux and laterally the basipetal flux (Roychoudhry and Kepinski 2022). The network of regulators controlling PIN vectorial transport activities is expanding since their original discovery by Wisniewska et al. (2006).

**FIGURE 3:**
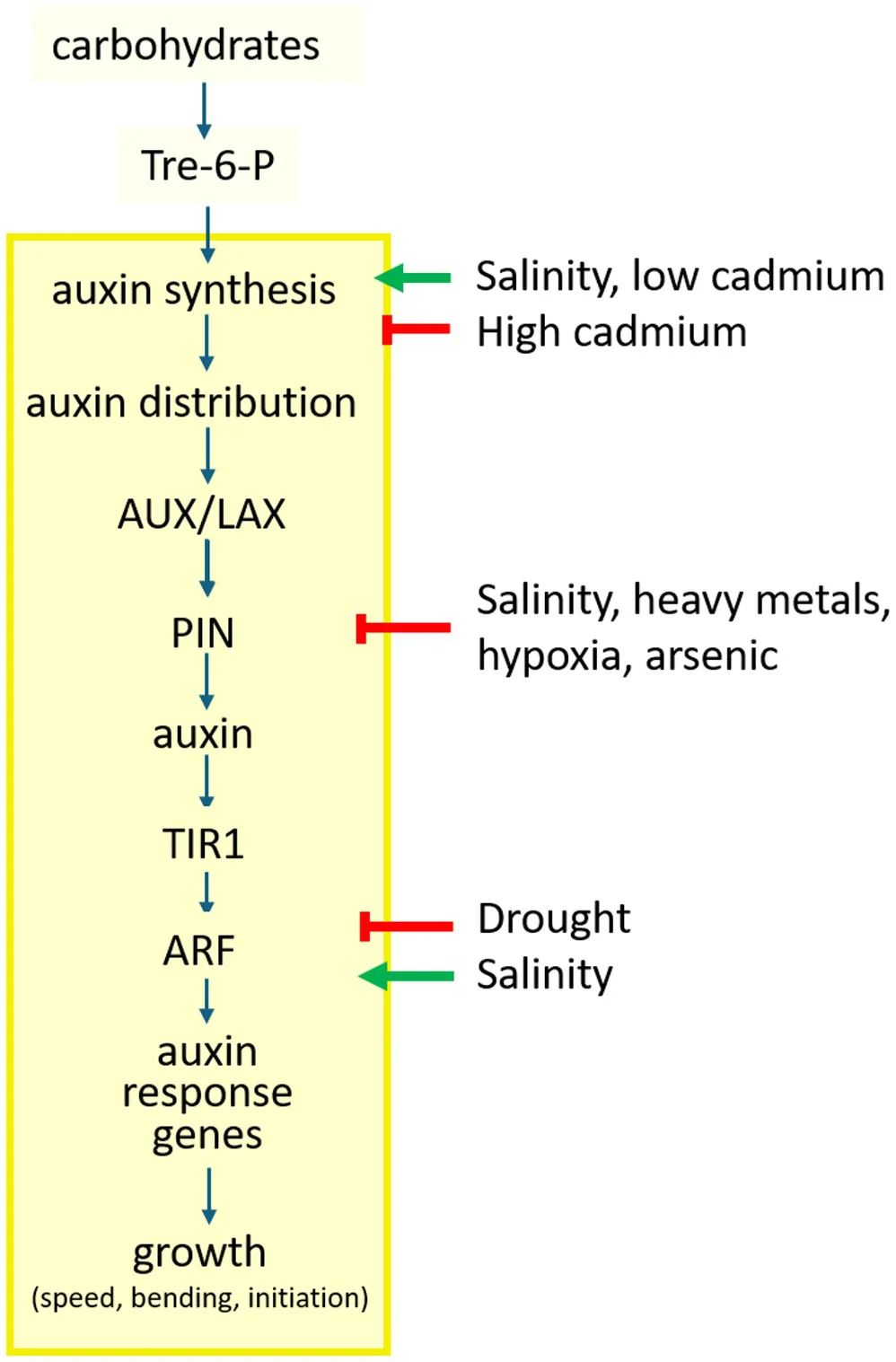
Simplified auxin‐dependent signaling pathway determining root growth. Also indicated is the input from carbon availability, that via the signaling molecule trehalose‐6‐phosphate initiates the auxin signaling pathway ultimately affecting root growth, lateral root initiation, and bending. Selected stress effects on components of the pathway are indicated based on Marzi et al. (2024).

Recently, an enhancing positive feedback mechanism has been described that involves auxin sensing by the AUXIN‐BINDING PROTEIN‐LIKE 3 (ABL3) and the TRANSMEMBRANE KINASE 1 (TMK1). Auxin binding to this complex leads to phosphorylation and stabilization of PIN2 and a concomitant asymmetric auxin flux for root bending (Rodriguez et al. 2025). The authors suggest that this mechanism is also involved in other self‐organizing developmental processes, although the mechanism of phosphorylation‐dependent regulation of PIN activity, polarity, and stability still awaits clarification. The vectorial transport of auxin in the roots is the major mechanism that converts environmental input into an adaptive root growth response and root system architecture (RSA).

Execution of the auxin signaling response involves the repressors of the AUXIN/INDOL‐3‐ACETIC ACID protein (AUX/IAA) family with 34 members in *A. thaliana* (Taylor and Bargmann 2024), the activating transcription factor family AUXIN RESPONSE FACTORS (ARF) with 23 members in *A. thaliana* (Guilfoyle and Hagen 2007), and the auxin receptors of the TRANSPORT INHIBITOR RESPONSE 1/AUXIN SIGNALING F‐BOX PROTEIN 1–5 (TIR1/AFB1‐5) family (Villalobos et al. 2012). The combinatorial richness of the sensor/execution system reflects the significance of the auxin response in plants and should be addressed in multifactorial stress environments (Villalobos et al. 2012; Taylor and Bargmann 2024; Vanneste et al. 2025).

The dynamic and flexible activation of the described stress sensing, transduction, and execution pathways enables the developmental plasticity of RSA and is a decisive part of the successful history of plants in settling on land (Morris et al. 2017). Detailed analysis of root responses to single environmental challenges has clarified the diverse and often contrasting responses of RSA, but there is a need to scrutinize stress combinations under controlled conditions. The following section focuses on one example, the combination of arsenic and hypoxic stress, where transcriptome analysis provides insight into the adjustment of the described regulatory network.

## 4 The Combinatorial Response of *Arabidopsis thaliana* Roots to Arsenic and Hypoxic Stress

The metalloid arsenic is a naturally occurring environmental hazard that reacts with thiols as As(III) or substitutes for phosphate as As(V) (Sharma et al. 2021). Arsenic (As) exposure causes oxidative stress, metabolic disturbance, and genetic mutations. Likewise, low O_2_ (hypoxia, Hpx) inhibits O_2_‐dependent respiration and necessitates metabolic rearrangements to adjust plant growth and survival to low energy provision (Renziehausen et al. 2025). Hpx often develops as a consequence of flooding during heavy rainfall. Arsenic intoxication depends on geological formations and human activities and is encountered in particular upon irrigation with As‐contaminated groundwater. In a series of studies, Kumar et al. (2019, 2020, 2026) scrutinized the single effect of either Hpx or As as well as the combined HpxAs stress on *A. thaliana* performance, both under acute stress and recovery.

In this experimental setup, Hpx was established by flushing the hydroponics medium with O_2_‐deprived air instead of normal ambient air. Under this condition, the O_2_ concentration decreases to 86 instead of 262 nmol O_2_ mL^−1^ in the medium aerated with ambient air, and this lowered O_2_^−^‐availability fully inhibits root growth. Importantly, recovery of roots from combined HpxAs stress is significantly delayed compared to single Hpx stress (Kumar et al. 2019). The treatment combination of HpxAs triggers severe metabolic changes both locally and, interestingly, also systemically, although Hpx was only established in the rooting medium (Kumar et al. 2019, 2026).

The specific HpxAs effects on root physiology and development comprise a stronger deregulation of redox homeostasis, as indicated by the recording of the in vivo glutathione redox sensor roGFP‐GRX. In parallel, the roots accumulate the plant growth regulator oxylipin 12‐oxophytodienoic acid (OPDA), show stimulated root hair growth and, upon reaeration, slower recovery from inhibition of apical growth (Kumar et al. 2019, 2020).

About 20% of the transcripts specifically deregulated in HpxAs have reported roles in root growth and root hair development (Kumar et al. 2019). A selection of transcriptional responses of root growth‐related genes in the single and combined treatments compared to untreated control roots is presented in Table 1 (extracted from Kumar et al. 2020). The majority of the transcripts involved in auxin transport and metabolism, signaling and cell fate respond similarly in Hpx and HpxAs. However, interesting exceptions from this rule concern the stronger increase in PILS1 transcript and the much stronger down‐regulation of the auxin efflux carriers LAX2 and LAX3 in HpxAs. Profound differences between Hpx and HpxAs are seen for the selected transcripts involved in root cell fate determination, for example, AIR1 controlling root hair development, whose abundance increases in Hpx but strongly decreases in HpxAs and even more in As (Table 1). Auxin target genes such as flavonol synthase 1 (FLS1), an enzyme downstream of auxin signaling, behave distinctly in HpxAs‐treated plants compared to Hpx and As.

**TABLE 1:** Response of the *Arabidopsis thaliana* root transcriptome to arsenic (As), hypoxia (Hpx), and arsenic‐hypoxia As/Hpx combined treatment (Kumar et al. 2020).

Name	AT‐number	Function	As/C	Hpx/C	AsHpx/C
Transport					
PIN1	AT1G73590	Auxin efflux carrier	−2.05*	−3.33*	−3.19*
PIN4	AT2G01420	Auxin efflux carrier	−1.01	−2.26*	−2.12*
PIN5	AT5G16530	Auxin efflux carrier	−1.04	1.19	1.29
PILS1	AT1G20925	PIN‐likes 1, auxin efflux	1.22	1.33	1.67°
PILS3	AT1G76520	Auxin transport facilitator	7.98*	1.15	1.48
PILS4	AT1G76530	Auxin transport facilitator	−1.19	1.04	1.40
ABCB22	AT3G28415	Basipetal auxin transport	1.32	2.61°	2.62*
ABCG37	AT3G53480	Auxin polar transport	4.02*	1.55°	1.84*
LAX2	AT2G21050	Auxin influx carrier	−2.20*	−5.55*	−9.73*
LAX3	AT1G77690	Auxin influx carrier	−2.31*	−2.03*	−2.77*
ZIFL1	AT5G13750	Basipetal auxin transport; root development	6.70*	−1.29	1.05
Cell fate					
WOX5	AT3G11260	Stem cell maintenance	−1.06	1.13	−1.11
WOX11	AT3G03660	Adventitious root development	1.28	6.21*	5.46*
AIR1	AT4G12550	Lateral root root hair development,elongation	−147.66*	2.35	−15.49*
AIR12	AT3G07390	Lateral root morphogenesis	1.31	−1.96	−2.82°
LBD29	AT3G58190	Lateral root formation	−1.72°	3.16*	−1.69
NOV	AT4G13750	Cell fate specification, stem cell maintenance	2.34*	−1.03	1.99*
PLT1	AT3G20840	QC specification and stem cell activity	−1.51	−1.24	−1.40
Signaling					
ARF3	AT2G33860	Response to auxin	−1.51*	−1.42	1.33
ARF5	AT1G19850	Response to auxin	−1.03	1.85	1.97°
ARF6	AT1G30330	Response to auxin	−1.09	−1.09	1.45
ARF11	AT2G46530	Response to auxin	1.14	1.10	1.35*
ARF12	AT1G34310	Response to auxin	1.23	1.69	1.55
MRF1	AT5G63190	Auxin‐/sugar‐activated signaling	−1.09	1.77*	2.47*
MPK12	AT2G46070	Signaling, response to auxin	−1.00	3.15*	5.17*
TIR1	AT3G62980	Auxin receptor	−1.17°	1.30	1.65
Auxin metabolism					
GH3	AT5G13370	Conjugates glutamine to IBA	12.79*	1.08	2.39*
GH3.2	AT4G37390	Indole‐3‐acetic acid‐amido synthetase	2.35*	2.32*	4.16
GH3.5	AT4G27260	IAA‐amido synthase	−1.40	1.55	1.36
TAR2	AT4G24670	Tryptophan aminotransferase related 2	−2.07*	−2.12	−3.63*
YUC4	AT5G11320	Indole‐3‐pyruvate monooxygenase	1.08	3.49*	2.91*
YUC9	AT1G04180	Auxin biosynthetic process	1.53	2.39*	2.05*
Downstream targets					
FLS1	AT5G08640	Response to auxin, flavonol synthase 1	10.57*	1.24	2.75*
IAA3	AT1G04240	Auxin‐responsive protein, lateral root growth	−1.31	−3.65*	−3.61*
IAA32	AT2G01200	Auxin‐responsive protein	1.22	1.87	3.01°
SAUR41	AT1G16510	Small auxin upregulated 41	1.35	2.69*	2.97*
SAUR48	AT3G09870	Small auxin upregulated 48	−1.00	1.61	1.49°
SAUR57	AT3G53250	Small auxin upregulated 57	1.20	−1.02	1.39°
SAUR59	AT3G60690	Small auxin upregulated 59	−1.30	1.67	1.40

*Note:* Focus is given to genes that regulate root development. The color code depicts fold‐change groups as indicated below. In addition * marks significance of difference to the control of *p* ≤ 0.05 and ^°^ *p* ≤ 0.1. 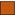 *x* ≤ −4, 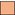 −4 < *x* ≤ −2.5, 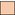 −2.5 < *x* ≤ −1.5, 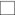 −1.5 < *x* < 1.5, 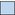 1.5 ≤ *x* < 2.5, 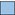 2.5 ≤ *x* < 4, 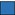 4 ≤ *x*.

Detailed analyses of transcripts involved in K‐homoeostasis, sulfur metabolism, and redox regulation confirm the severity of the combined HpxAs stress compared to the single stressors as well (Kumar et al. 2019). Thus, the transcriptome analyses provide tentative clues on the differential response of roots to single and combined environmental constraints and the involved regulatory mechanisms.

## 5 Combined Nutrient Deficiency Effects on Barley Root Exudation

Apart from direct effects of stressors on regulatory mechanisms controlling RSA and root physiology, single and combinatorial environmental factors interfere with the root × environment interface. Roots exude metabolites and ions into the substrate and, thereby, shape the composition of the rhizosphere (Wankhade et al. 2025; Adeniji et al. 2025). Exudation represents a small part of rhizodeposition which comprises release and decay of dead root cells including root hairs, transfer of nutrients to mycorrhiza and exudation (Figure 4). Apart from small lipophilic compounds that can freely diffuse through biomembranes, various types of transporters catalyze the metabolite transfer, for example, SWEET for sugars (Loo et al. 2024), UMAMIT for amino acids (Kim et al. 2021), ABC for various specialized metabolites (Badri et al. 2008) and MATE for organic acids (Delhaize et al. 2007).

**FIGURE 4:**
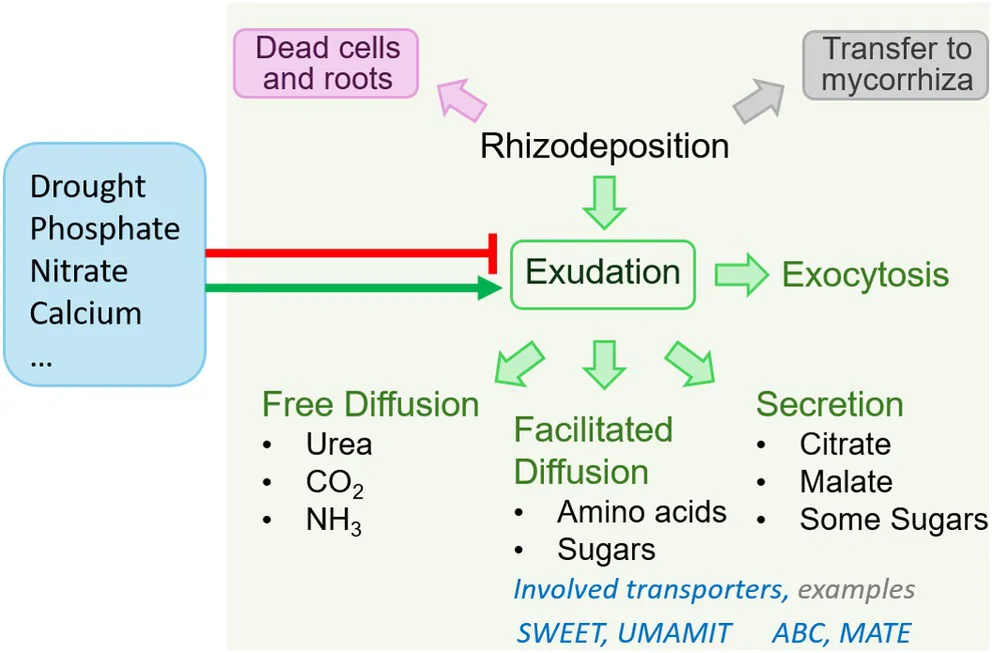
Overview of rhizodeposition. Roots deliver material to the rhizosphere via release of dead cells, material transfer to mycorrhiza, and transport across the plasmamembrane via exudation and exocytosis. Three types of transport mechanisms are involved that use different molecular driving forces and release different compounds. Four examples of involved transporters are indicated. Also indicated are examples of environmental parameters that interfere with root exudation.

Exuded organic acids, phenolics, and other chelators ease solubilization and acquisition of mineral nutrients such as iron and phosphate, assist in nitrogen cycling, and ameliorate aluminum toxicity by external immobilization (Chai and Schachtman 2022). Exudation is flexibly and dynamically adjusted to nutrient availability, abiotic and biotic conditions, and local gradients and hotspots in heterogeneous soil. Released strigolactones assist in recruiting microorganisms and facilitate nutrient acquisition (Santoro et al. 2021; Kim et al. 2022). Specialized metabolites such as artemisin are suggested to modulate allelopathic and symbiotic interactions in the rhizosphere (Paponov et al. 2023). Exudation patterns strongly vary among species and genotypes (Giles et al. 2017).

A recent study explored the effect of nitrogen deprivation (—N), calcium deficiency (—Ca), and combined stress imposed by withholding both N and Ca (—N/—Ca) on barley root exudation (Denjali et al. 2026). Sugar release immediately stops upon transfer to N‐deficient and —N/—Ca medium, as measured by collecting the exudates for the first 6 h after transfer to the deprived media (Denjali et al. 2026). In a converse manner, amino acid exudation under —N is maintained if combined with —Ca, indicating a role for Ca in the fast suppression of amino acid exudation under —N (Denjali et al. 2026). Strongly enhanced amino acid exudation under —N in the presence of Ca‐channel blockers lanthanum and verapamil relative to the untreated roots supports the conclusion of a major role of Ca in the inhibition of amino acid exudation under —N (Denjali et al. 2026). On the longer time scale of 10 d, the metabolome analysis of the exudate revealed specificity in the response of different amino acids, with the biggest difference in release between —N and —N/—Ca for isoleucine and serine.

Other abiotic stresses also strongly affect root exudation, for example, water deficit stimulates release of many amino acids, in particular serine, aspartate, asparagine, glutamic acid and tryptophan in cotton during an 11‐day aeroponic treatment (Coker et al. 2024). Maize roots also stimulate glycine and alanine exudation upon dehydration, and also under heat, and this response is roughly additive under combined drought and heat treatment (Tiziani et al. 2022).

Since root exudates participate in shaping the rhizosphere by interfering with the rhizobiome, including the root association of beneficial plant growth‐promoting microorganisms (Lugtenberg and Kamilova 2009; Liu et al. 2025), the interaction of single and combinatorial environmental factors on root exudation processes should receive more attention both in terms of fundamental understanding and application, since tailoring the microbiome promises novel or refined approaches in counteracting negative effects of abiotic and biotic stresses in agriculture (Copeland et al. 2025; Salse et al. 2024).

## 6 Triple Combinations of Environmental Stress on Sugar Beet Roots

Dual combinations of environmental stressors are a first step in bridging laboratory experiments and field studies. Ron Mittler's group has pioneered research in the field of multifactorial stress effects on plants and alga (Zandalinas, Casal, et al. 2024; Zandalinas, Peláez‐Vico, et al. 2024; Pascual et al. 2025). These important studies mostly addressed the performance of the shoot. García‐Caparrós et al. (2022) focused on the roots in barley and combined two soil parameters, namely temperature and salinity, with the systemic signal of low and high photosynthetic irradiation, modifying resource allocation to the root. The combination of salinity and high temperature increases the C/N ratio in measured root metabolites despite receiving full nutrient supply. This effect is exacerbated during the high light treatment and accompanied by increased root energization and decreasing levels of phosphorylated metabolites of central metabolism (García‐Caparrós et al. 2022). The authors concluded that the combined stress imposes limitations on sucrolysis and central metabolism.

## 7 The Challenge of Combinatorial Stress Analyses of Root Growth

Experimental studies aiming at understanding the root responses to combinatorial stresses should include the single stress treatments and the lower‐order combinations as well. This necessity exhausts the experimental capacity of most laboratories and budgets, since the analysis of three parameters already comprises a setup of 8 treatments, four parameters 16 conditions and five parameters 32 conditions. And despite the exponential increase in sample number, the analysis with this sampling design only explores single quantities of the selected stressors and a single time point of harvest. Importantly, plants are exposed to virtually infinite combinations of environmental factors, considering the diversity of environmental chemical and physical parameters, the concentration and severity range, the order of impact, the spatial heterogeneity for example, in the soil, and the previous individual history of the plant establishing stress memory (Kumar et al. 2024; Aswathi et al. 2025).

The chemical and physical heterogeneity at various spatial scales is an intriguing characteristic of soils and poses a particular challenge to plants and researchers. Salinity gradients stimulate root growth away from the higher salt concentration, a response termed halotropism (Galvan‐Ampudia et al. 2013). Asymmetric endocytosis eliminates PIN2 from the plasmamembrane exposed to the higher NaCl concentration. Subsequent establishment of higher auxin concentrations on the distant side stimulates cell expansion and bending of the roots to the area of the rooting substrate with less NaCl.

## 8 Summary and Outlook

The described case studies exemplify experimental investigations that have deepened our understanding of root responses to multifactorial abiotic stresses. The information covered here cannot be comprehensive due to space limitations. However, based on the available literature, a first conclusion is that multifactorial stress effects on roots have been investigated more frequently than commonly thought and stated in many current publications. The majority of published studies describe growth phenomena and changes in physiological parameters in the context of plant mineral nutrition and yield formation. To a much lower extent, the studies explore the underlying molecular mechanisms of stress perception and signal integration.

### 8.1 Identifying Integrating Hubs, Involved Pathways and Central Networks

Understanding the root response to multifactorial stress relies on identifying the regulatory pathways and interaction network(s) that sense, transmit, and integrate the stress inputs and tune the root response (Figure 5). The understanding is quite advanced for laboratory experiments applying single stresses. Hubs and bottlenecks are central elements in the crosstalk of signaling networks that shape the cellular responses (Dietz et al. 2010) and serve as modules of signal integration in multifactorial stress combinations (Kumar et al. 2024, 2025). An example of such an integrating module is the TORC1 complex with multiple input parameters from C—, N—, S—, and energy metabolism. Carbohydrates, auxin, and redox cues play decisive roles in adjusting RSA to environmental conditions and interact with specific sensory systems such as nitrate sensing, but also with the pathways of other plant hormones.

**FIGURE 5:**
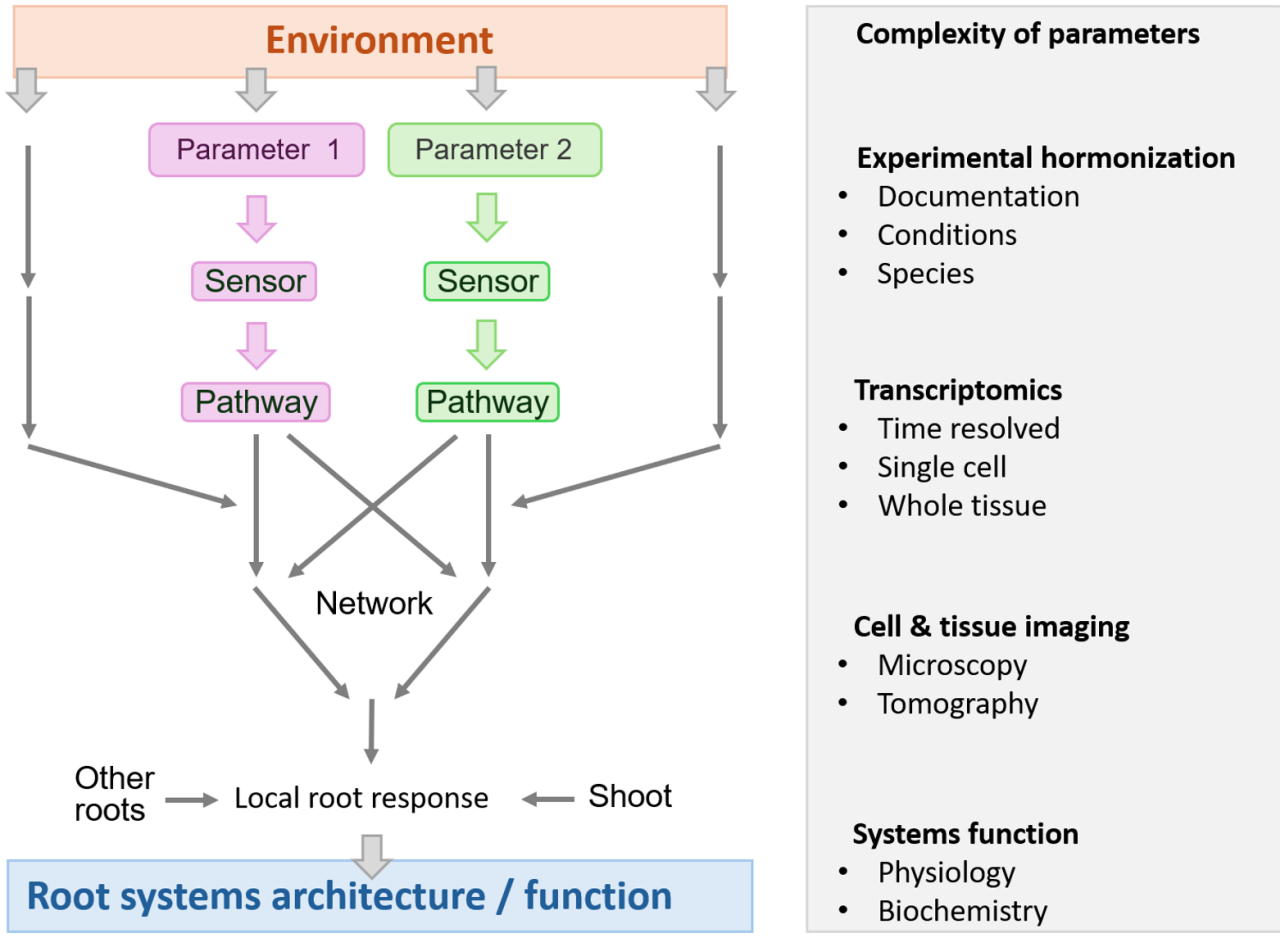
Simplified schematics of the link between environmental parameters and the local root growth response. Diverse parameters from the environment initiate signaling via sensors and pathways that crosstalk in networks. The network integrates the inputs and regulates the local root growth response. Additional inputs systemically derive from other roots and the shoot. Research perspectives are indicated on the right hand side that are expected to advance our understanding of multifactorial stress acclimation of roots in the future.

### 8.2 Tissue Transcriptomes as First Proxy Toward Identification of Regulatory Mechanisms

For the time being, transcriptome analyses by RNA sequencing provide an accessible and affordable approach to study the interactions of multiple factors. What we need is an initiative for stress combinations similar to the ATGenExpress two decades ago that coordinated growth and experimental regimes, collected Gene Chip based transcriptomes on single stress acclimatization and provided the data to the community of plant researchers (Kilian et al. 2007). The research field of multifactorial acclimatization and adaptation at present is fragmented and heterogeneous and would benefit from attempts at harmonizing documentation, experimental conditions and the use of species.

### 8.3 The Inevitable Reductionism of Current Experimental Designs

Current drawbacks in analytical strategies emerge from the frequent focus on a single time point of harvest and the selective scrutiny of usually a unique pair of combinatorial stress intensities. Multifactorial stresses do not elicit linear responses but rather evoke a complex multidimensional acclimatization pattern. This complexity must be brought into the focus of research.

### 8.4 Local Decisions Define the RSA and Root Function

On top of these limitations, root development, as pointed out in this review, is the result of localized regulation of cell and tissue activities in the RAM for root hair formation, initiation of lateral and adventitious roots, and so on. Global transcriptomes often fail to provide access to these spatially and temporally triggered responses. Sorting and analysis of defined cell types are needed to understand the spatially confined molecular regulation and should be combined with detailed dissection of the activity and fate of specific cells and tissues by modern microscopy, tomography, and other phenotyping approaches.

### 8.5 The Aspect of Soil Heterogeneity

Lastly, another important aspect of RSA development concerns the heterogeneity of soil and the required local responses of roots to beneficial and deleterious conditions. The local responses, however, are part of the whole plant acclimatization process, and the coordination and optimization of the response depend on systemic signaling and material exchange with other roots and the shoot (Figure 5).

## Author Contributions

Karl‐Josef Dietz conceived the review, analyzed the data, and wrote the manuscript.

## Funding

This work was supported by Deutsche Forschungsgemeinschaft, DI 346/23‐1.

## Disclosure

The paper and the figures are designed without the use of generative AI.

## Conflicts of Interest

The author declares no conflicts of interest.

## Data Availability

This review does not report new data.
